# Kinetic Evaluation of Photosensitivity in Bi-Stable Variants of Chimeric Channelrhodopsins

**DOI:** 10.1371/journal.pone.0119558

**Published:** 2015-03-19

**Authors:** Shoko Hososhima, Seiichiro Sakai, Toru Ishizuka, Hiromu Yawo

**Affiliations:** 1 Department of Developmental Biology and Neuroscience, Tohoku University Graduate School of Life Sciences, Sendai, Japan; 2 Core Research of Evolutional Science & Technology (CREST), Japan Science and Technology Agency (JST), Tokyo, Japan; 3 Laboratory for Local Neuronal Circuits, Brain Science Institute, RIKEN, Wako, Japan; 4 Center for Neuroscience, Tohoku University Graduate School of Medicine, Sendai, Japan; University at Buffalo, UNITED STATES

## Abstract

Channelrhodopsin-1 and 2 (ChR1 and ChR2) form cation channels that are gated by light through an unknown mechanism. We tested the DC-gate hypothesis that C167 and D195 are involved in the stabilization of the cation-permeable state of ChRWR/C1C2 which consists of TM1-5 of ChR1 and TM6-7 of ChR2 and ChRFR which consists of TM1-2 of ChR1 and TM3-7 of ChR2. The cation permeable state of each ChRWR and ChRFR was markedly prolonged in the order of several tens of seconds when either C167 or D195 position was mutated to alanine (A). Therefore, the DC-gate function was conserved among these chimeric ChRs. We next investigated the kinetic properties of the ON/OFF response of these bi-stable ChR mutants as they are important in designing the photostimulation protocols for the optogenetic manipulation of neuronal activities. The turning-on rate constant of each photocurrent followed a linear relationship to 0–0.12 mWmm^−2^ of blue LED light or to 0–0.33 mWmm^−2^ of cyan LED light. Each photocurrent of bi-stable ChR was shut off to the non-conducting state by yellow or orange LED light in a manner dependent on the irradiance. As the magnitude of the photocurrent was mostly determined by the turning-on rate constant and the irradiation time, the minimal irradiance that effectively evoked an action potential (threshold irradiance) was decreased with time only if the neuron, which expresses bi-stable ChRs, has a certain large membrane time constant (eg. τ_m_ > 20 ms). On the other hand, in another group of neurons, the threshold irradiance was not dependent on the irradiation time. Based on these quantitative data, we would propose that these bi-stable ChRs would be most suitable for enhancing the intrinsic activity of excitatory pyramidal neurons at a minimal magnitude of irradiance.

## Introduction

Many living organisms use light as a carrier of information through converting photon energy to a biochemical or biophysical signal. In the case of the unicellular green algae *Chlamydomonas reinhardtii*, photon energy is captured by two channelrhodopsins (ChRs), ChR1 and ChR2, which are localized in small regions of the plasma membrane covering the eyespot, and is conducted to the cell as an electrical signal in the membrane [1], [2], [3], [4]. Each ChR is a member of the microbial-type rhodopsin family and has a seven-pass transmembrane (TM) apoprotein with a covalently bound retinal [5], [6], [7]. In the case of ChR2, the basal state with all-*trans* retinal (D480) is non-conductive to any ions. Light absorption is followed by the photoisomerization of the all-*trans* retinal to a 13-*cis* configuration and drives cyclic conformational changes of the molecule, called photocycles, which consist of several intermediates such as P390 and at least two states conductive to cations [8], [9], [10], [11], [12], [13]. Consequently, very rapid (in the orders of ms) generation of a photocurrent is induced in cell membranes expressing ChR2 [1], [2], [14], [15].

Recently it has been experimentally demonstrated that the P520 conductive state is stabilized by the introduction of point mutations in C128 and/or D156 of ChR2 [16], [17], [18]. These residues are conserved among many ChRs, but with some exceptions [7], [19]. These two amino acids are suggested to form a structural motif named DC gate as their arrangement would form the molecular switch that determines the transition from a conductive to non-conductive state [13], [20], [21]. The counterparts of these amino acid residues are C167 and D195 in ChRWR/C1C2, a chimeric ChR consisting of TM1-5 of ChR1 and TM6-7 of ChR2 [22], [23]. The crystallographic study indicated that C167 lies close to all-*trans* retinal with some interaction, but has a form rather less interactive with D195 at the basal state [24]. Here we tested the hypothesis that these residues are also involved in the stabilization of the conductive state of ChRWR. The results suggest that the conductive state of ChRWR or ChRFR, another chimeric ChR consisting of TM1-2 of ChR1 and TM3-7 of ChR2 [22], is indeed stabilized by a mutation of either C167 or D195.

Those ChR variants, such as ChR2-C128S, have been referred to as bi-stable or step-function mutants as they are turned on to the conductive state (ON response) by blue light and become non-conductive by light (OFF response) of another wavelength [16], [17], [18]. It has been generally difficult to study the ON response because the individual molecules are photocycling rapidly and asynchronously. The bi-stable ChRs are ideal to investigate the ON and OFF responses as functions of irradiance and time because these two processes are independent, although only limited analyses have been performed so far [16], [17], [18]. A quantitative description of the kinetic properties is also necessary for designing the stimulation protocols, as these ChRs have been recently applied to enhance the neuronal and glial activities *in vivo* [18], [25], [26], [27], [28]. In this paper, we revealed each ON/OFF response as a function of irradiance and time. Based on these quantitative data, we would propose that these bi-stable ChRs would be most suitable for enhancing the intrinsic activity of excitatory pyramidal neurons at a minimal magnitude of irradiance.

## Materials and Methods

### Animals

All animal experiments were approved by the Tohoku University Committee for Animal Experiments (Approval No. 2013LsA-016) and were carried out in accordance with the Guidelines for Animal Experiments and Related Activities of Tohoku University as well as the guiding principles of the Physiological Society of Japan and the National institutes of health (NIH), USA. The number of animals in this study was kept to a minimum and, when possible, all animals were anesthetized to minimize their suffering.

### Cell culture and molecular biology

The transfection plasmid vectors were made as described previously [22] containing a C-terminal fusion construct with *Venus* [29] of either ChR2(1–315), ChRWR/C1C2, which consists of the TM1-5 of ChR1 and the TM6-7 of ChR2, or ChRFR, which consists of the TM1-2 of ChR1 and the TM3-7 of ChR2. Amino acid substitutions of these ChRs were introduced by PCR-based site-directed mutagenesis using the KOD -Plus- Mutagenesis Kit (Toyobo, Osaka, Japan). All PCR-derived constructs were verified by sequencing.

The electrophysiological assays of ChR variants were made using ND7/23 cells, hybrid cell lines derived from neonatal rat dorsal root ganglia neurons fused with the mouse neuroblastoma [30]. ND7/23 cells were grown in Dulbecco's modified Eagle’s medium (Wako, Osaka, Japan) supplemented with 10% fetal bovine serum under a 5% CO_2_ atmosphere at 37°C. The expression plasmids were transiently transfected in ND7/23 cells using Effectene Transfection Reagent (Qiagen, Tokyo, Japan) according to the manufacturer’s instructions. Electrophysiological recordings were then conducted 16–48 h after the transfection. Successfully transfected cells were identified by the presence of Venus fluorescence.

Cortical neurons were isolated from embryonic day 16 Wistar or Sprague-Dawley rats (Japan SLC Inc., Shizuoka, Japan) using Nerve Cell Dissociation Medium (Sumitomo Bakelite, Tokyo, Japan) according to the manufacturer's instructions and grown in culture medium (Sumitomo Bakelite, Tokyo, Japan) under a 5% CO_2_ atmosphere at 37°C. The expression plasmids were transiently transfected in cortical neurons calcium phosphate transfection at days *in vitro* (DIV) 5 or 6. Electrophysiological recordings were then conducted at DIV 21–23 to neurons identified to express Venus fluorescence under conventional epifluorescence system.

### Electrophysiology

All experiments were carried out at room temperature (23 ± 2°C). Photocurrents were recorded as previously described [15] using an EPC-8 amplifier (HEKA Electronic, Lambrecht, Germany) under a whole-cell patch clamp configuration. The data were filtered at 1 kHz and sampled at 10 kHz (Digdata1440 A/D, Molecular Devices Co., Sunnyvale, CA) and stored in a computer (pClamp10.3, Molecular Devices). The absence of dye coupling was confirmed by visualization of Alexa Fluor 568 (Life Technologies, Carlsbad, California, USA).

The internal pipette solution for whole-cell voltage-clamp recordings from ND7/23 cells contained (in mM) 120 KOH, 100 glutamate, 5 EGTA, 20 HEPES, 2.5 MgCl_2_, 2.5 Mg-ATP, 0.1 Leupeptin, 0.01 Alexa Fluor 568, adjusted to pH 7.3 with KOH. The internal pipette solution for the whole-cell current-clamp recordings from cortical neurons contained (in mM) 125 K-gluconate, 10 KCl, 0.2 EGTA, 10 HEPES, 1 MgCl_2_, 3 Mg-ATP, 0.3 Na_2_GTP, 10 Na_2_-phosphocreatine, 0.1 Leupeptin, adjusted to pH 7.2 with KOH. The extracellular ACSF solution contained (in mM) 125 NaCl, 2.5 KCl, 25 NaHCO_3_, 1.25 NaH_2_PO_4_, 2 CaCl_2_, 1 MgCl_2_, 11 glucose, bubbled with mixed gas containing 95% O_2_ and 5% CO_2_. In all cortical neuron experiments, ACSF contained 20 μM 6,7-Dinitroquinoxaline-2,3-dione (DNQX, Tocris Bioscience, Ellisville, Missouri, USA), 25 μM d-(−)-2-amino-5-phosphonovaleric acid (d-AP5, Tocris), and 100 μM picrotoxin (Nacalai, Kyoto, Japan) to block all synaptic inputs.

### Optics

Irradiation was carried out using power LEDs (each from Philips Lumileds Lighting Inc., San Jose, CA) emitting either blue light (peak, 460–490 nm, LXHL-NB98,), cyan light (peak, 490–520 nm, LXML PE01-0070), yellow light (peak, 587–597 nm, LXHL-NL98) or orange light (peak, 612–620nm, LXM2 PH01-0070) controlled by a regulator (SLA-1000-2, Mightex, Toronto, Canada) and computer software (pCLAMP10.3, Molecular Devices). The power of LED light was directly measured under microscopy by a visible light-sensing thermopile (MIR-100Q, Mitsubishi Oil Chemicals, Tokyo, Japan) and divided by the irradiation area (0.16 mm^2^) to obtain irradiance (light power density).

The photocurrent amplitude and kinetics are dependent on the irradiance [15], the holding potential [1], [2], [15] and the pH [1], [2], [10]. Therefore, every photocurrent was measured with a holding potential of −60 mV and at pH 7.4 outside. Each ON/OFF photocurrent was fitted by a single-exponential function of the time during the transition phase between 10 and 90% of the peak response without any obvious deviation from the raw data.

### Statistical analysis

All data in the text and figures are expressed as mean ± SEM and were evaluated with the Mann-Whitney *U* test for statistical significance, unless otherwise noted. It was judged as statistically insignificant when P > 0.05.

## Results

### Effects of C167 or D195 mutation

**Fig 1:**
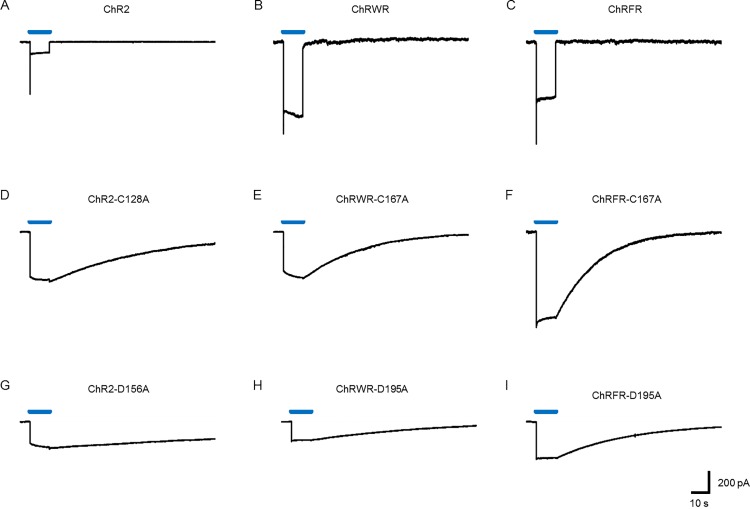
Photocurrent time course of chimeric ChRs and their DC gate mutants. Each trace is a typical photocurrent evoked by blue LED light (0.12 mWmm^−2^) for the time indicated by a blue line (10 s). **A,** ChR2. **B,** ChRWR/C1C2. **C,** ChRFR. **D,** ChR2-C128A. **E,** ChRWR-C167A. **F,** ChRFR-C167A. **G,** ChR2-D156A. **H,** ChRWR-D195A. **I,** ChRFR-D195A.

Targeted mutation of C167 of ChRWR was made by replacement of alanine (ChRWR-C167A) and compared with the counterpart mutation of ChR2 (ChR2-C128A) or ChRFR (ChRFR-C167A). As shown in Fig. 1, the mutation at this position markedly prolonged the OFF phase of the photocurrent of ChR2 (Fig. 1A vs. 1D), ChRWR (Fig. 1B vs. 1E) and ChRFR (Fig. 1C vs. 1F). Similarly the targeted mutation of D195 or its counterpart position prolonged the OFF phase of the photocurrent of ChR2 (ChR2-D156A, Fig. 1G), ChRWR (ChRWR-D195A, Fig. 1H) and ChRFR (ChRFR-D195A, Fig. 1I).

**Fig 2:**
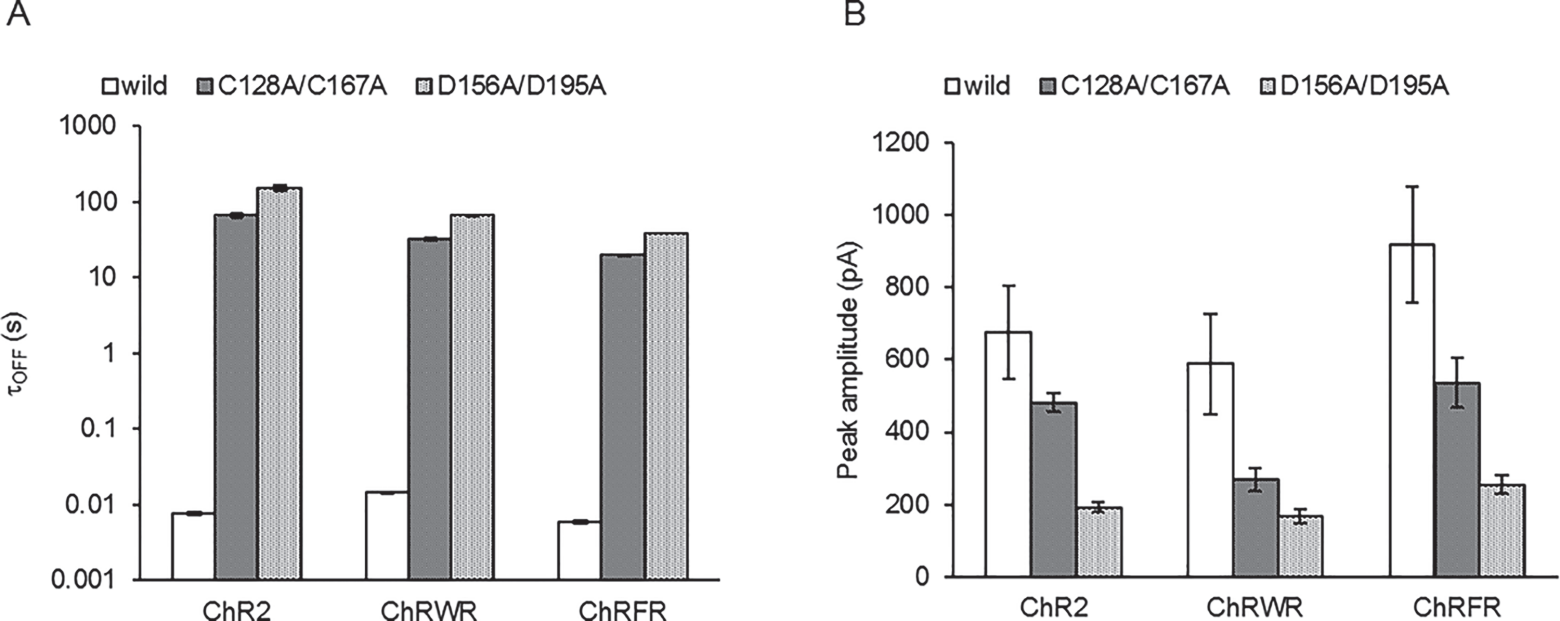
Summary of the effects of C128A/C167A or D156A/D195A mutation on the photocurrents of ChR2, ChRWR and ChRFR. **A,** Comparison of τ_OFF_. **B,** Comparison of peak amplitude.

Each OFF phase followed a single exponential relationship with a time constant (τ_OFF_) and is summarized in Fig. 2A. The τ_OFF_ of ChR2 was prolonged about 9,000-fold by the C128A mutation and about 20,000-fold by the D156A mutation. Those of ChRWR and ChRFR were prolonged about 2,000-fold and 3,000-fold, respectively by the C167A mutation and about 5,000-fold and 6,000-fold, respectively by the D195A mutation. The mutation also affected the photocurrent amplitude (Fig. 2B).

### Bi-stability of C167 mutants

**Fig 3:**
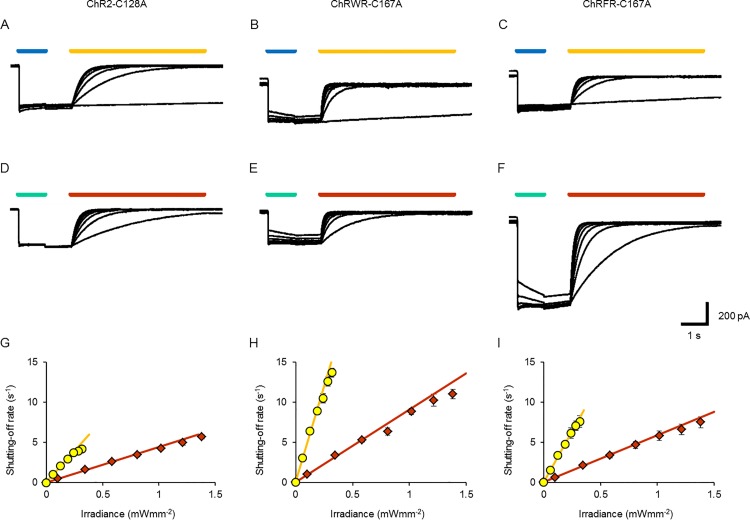
OFF response kinetics. **A–C,** Sample photocurrent records of ChR2-C128A (**A**), ChRWR-C167A (**B**) and ChRFR-C167A (**C**) opened by blue LED light (0.12 mWmm^−2^) and closed by yellow LED light (0.058–0.32 mWmm^−2^). **D-F,** Photocurrents of each bi-stable ChR opened by cyan LED light (0.33 mWmm^−2^) and closed by orange LED light (0.10–1.4 mWmm^−2^). **G,** Shutting-off rate constant (τ_OFF_^−1^) of ChR2-C128A as a function of irradiance by yellow LED light (yellow circles) and orange LED light (orange diamonds). Each line was fitted for the least-squares protocol; *y* = 16*x*+0.073 (yellow) and *y* = 4.4*x*+0.058 (orange). **H,** Similar relationships in ChRWR-C167A; *y* = 47*x*+0.23 (yellow) and *y* = 9.0*x*+0.12 (orange). **I,** Similar relationships in ChRFR-C167A; *y* = 25*x*+0.08 (yellow) and *y* = 5.8*x*+0.070 (orange).

The DC-gate mutant molecules of ChR2, such as ChR2-C128A, were turned on to the ion-conducting state by blue light and shut off to the non-conducting state by light of longer wavelengths [16] (Fig. 3A). Quite similarly, ChRWR-C167A and ChRFR-C167A were turned on by the blue LED light (peak, 460–490 nm) and shut off by the yellow LED light (peak, 587–597 nm) in a manner dependent on the irradiance (Fig. 3B and 3C). They were also turned on by the cyan LED light (peak, 490–520 nm) and shut off by the orange LED light (peak, 612–620 nm) (Fig. 3D-F). For each C167 variant, the shutting-off rate constant (τ_OFF_^−1^) was linearly dependent on the irradiance of either yellow LED light or orange LED light, as summarized in Fig. 3G (ChR2-C128A: slope, 16 s^−1^(mWmm^−2^)^−1^ for yellow LED light and 4.4 s^−1^(mWmm^−2^)^−1^ for orange LED light), Fig. 3H (ChRWR-C167A: slope, 47 s^−1^(mWmm^−2^)^−1^ for yellow LED light and 9.0 s^−1^(mWmm^−2^)^−1^ for orange LED light) and Fig. 3I (ChRFR-C167A: slope, 25 s^−1^(mWmm^−2^)^−1^ for yellow LED light and 5.8 s^−1^(mWmm^−2^)^−1^ for orange LED light). The ratio of these slopes by the changes of orange/yellow LED light (O/Y ratio) was 0.28 (ChR2-C128A), 0.19 (ChRWR-C167A) and 0.23 (ChRFR-C167A), respectively.

**Fig 4:**
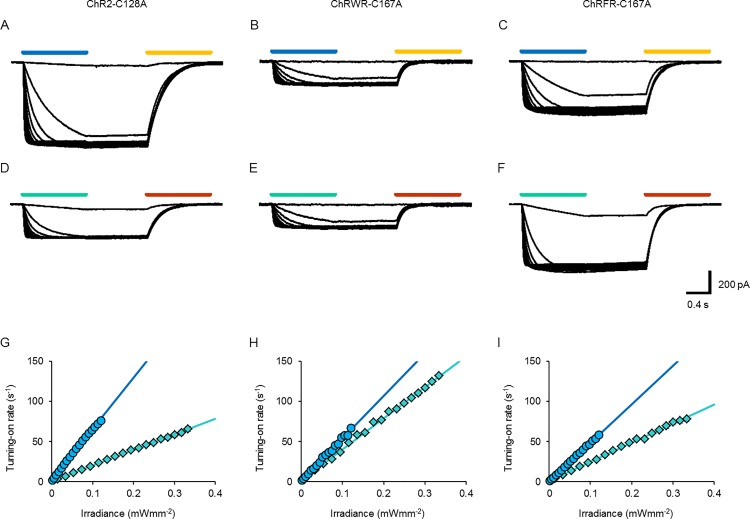
ON response kinetics. **A–C,** Sample photocurrent records of ChR2-C128A (**A**), ChRWR-C167A (**B**) and ChRFR-C167A (**C**) opened by blue LED light (0.0021–0.12 mWmm^−2^) and closed by yellow LED light (0.32 mWmm^−2^). **D-F,** Photocurrents of each bi-stable ChR opened by cyan LED light (0.014–0.33 mWmm^−2^) and closed by orange LED light (1.4 mWmm^−2^). **G,** Turning-on rate constant (τ_ON_^−1^) of ChR2-C128A as a function of irradiance by blue LED light (blue circles) and cyan LED light (cyan diamonds). Each line was fitted for the least-squares protocol; *y* = 640*x*+2.0 (blue) and *y* = 190*x*+0.76 (cyan). **H,** Similar relationships in ChRWR-C167A; *y* = 530*x*+1.1 (blue) and *y* = 390*x*+1.9 (cyan). **I,** Similar relationships in ChRFR-C167A; *y* = 480*x*+0.35 (blue) and *y* = 240*x*+1.1 (cyan).

As each light-dependent state transition of ChR is approximated by a single-photon reaction, each transition rate is presumed to be proportional to the irradiance. To test this, the photocurrents were evoked with varying powers of blue LED light (Fig. 4A-C) or cyan LED light (Fig. 4D-F) and the turning-on time constants (τ_ON_) were compared. Indeed, the turning-on rate constant (τ_ON_^−1^) of each photocurrent followed a linear relationship in a wide range of irradiance with either blue or cyan LED light, as summarized in Fig. 4G (ChR2-C128A: slope, 636 s^−1^(mWmm^−2^)^−1^ for blue LED light and 193 s^−1^(mWmm^−2^)^−1^ for cyan LED light), Fig. 4H (ChRWR-C167A: slope, 530 s^−1^(mWmm^−2^)^−1^ for blue LED light and 387 s^−1^(mWmm^−2^)^−1^ for cyan LED light) and Fig. 4I (ChRFR-C167A: 480 s^−1^(mWmm^−2^)^−1^ for blue LED light and 237 s^−1^(mWmm^−2^)^−1^ for cyan LED light). When the ratio of these slopes was calculated by the changes of cyan/blue LED light (C/B ratio), it was 0.30 (ChR2-C128A), 0.73 (ChRWR-C167A) and 0.49 (ChRFR-C167A), respectively.

### Regulation of neuronal excitability

**Fig 5:**
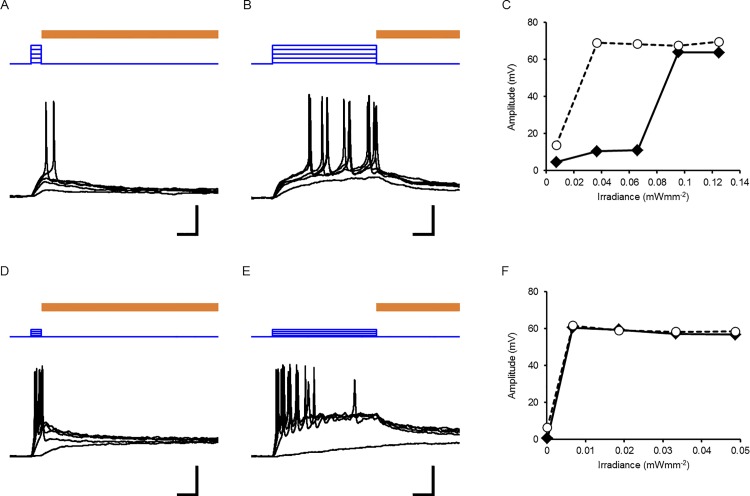
Photostimulation of a neuron as a function of time and irradiance. **A,** A series of recordings from a typical type-1 neuron. Depolarization by blue LED irradiation for 0.1 s (blue traces) and repolarization by yellow LED light for 5 s (brown line; 0.32 mWmm^−2^). The threshold irradiance was 0.095 mWmm^−2^. **B,** Another series of recordings from the same neuron with blue LED irradiation for 1 s with a threshold irradiance of 0.037 mWmm^−2^. **C,** The maximal depolarization amplitude as function of irradiance; closed diamond, 0.1-s pulse and open circle, 1-s pulse). **D,** Responses of a typical type-2 neuron to blue LED irradiation for 0.1 s (blue traces) with a threshold irradiance of 0.0066 mWmm^−2^. **E,** Responses to blue LED irradiation for 1 s (blue traces) with the same threshold irradiance. **F,** The maximal depolarization amplitude as a function of irradiance. Scale bars, 0.2 s (time), 0.2 mWmm^−2^ (blue traces: irradiance) and 20 mV (black traces: membrane potential) for A, B, D and E.

As the τ_OFF_^−1^ of each alanine-replaced mutant was negligible during blue/cyan irradiation, the magnitude of the photocurrent was almost determined by τ_ON_^−1^ and the irradiation time. Therefore, even a light in weak power can be expected to depolarize the neuronal membrane effectively to evoke action potentials with prolongation of the irradiation time. This idea was tested using cultured rat cortical neurons that expressed ChRFR-C167A. In a typical neuron, as shown in Fig. 5A, the minimal irradiance that effectively evoked an action potential (threshold irradiance) was 0.095 mWmm^−2^ when blue LED was turned on for 0.1 s. On the other hand, the threshold irradiance was as small as 0.037 mWmm^−2^ with 1-s pulse (Fig. 5B and 5C) in the same neuron. However, in another neuron (Fig. 5D-F), the threshold irradiance was the same between 0.1-s and 1-s pulses. This difference was attributed to the neuron’s intrinsic properties such as the membrane time constant (τ_m_).

**Fig 6:**
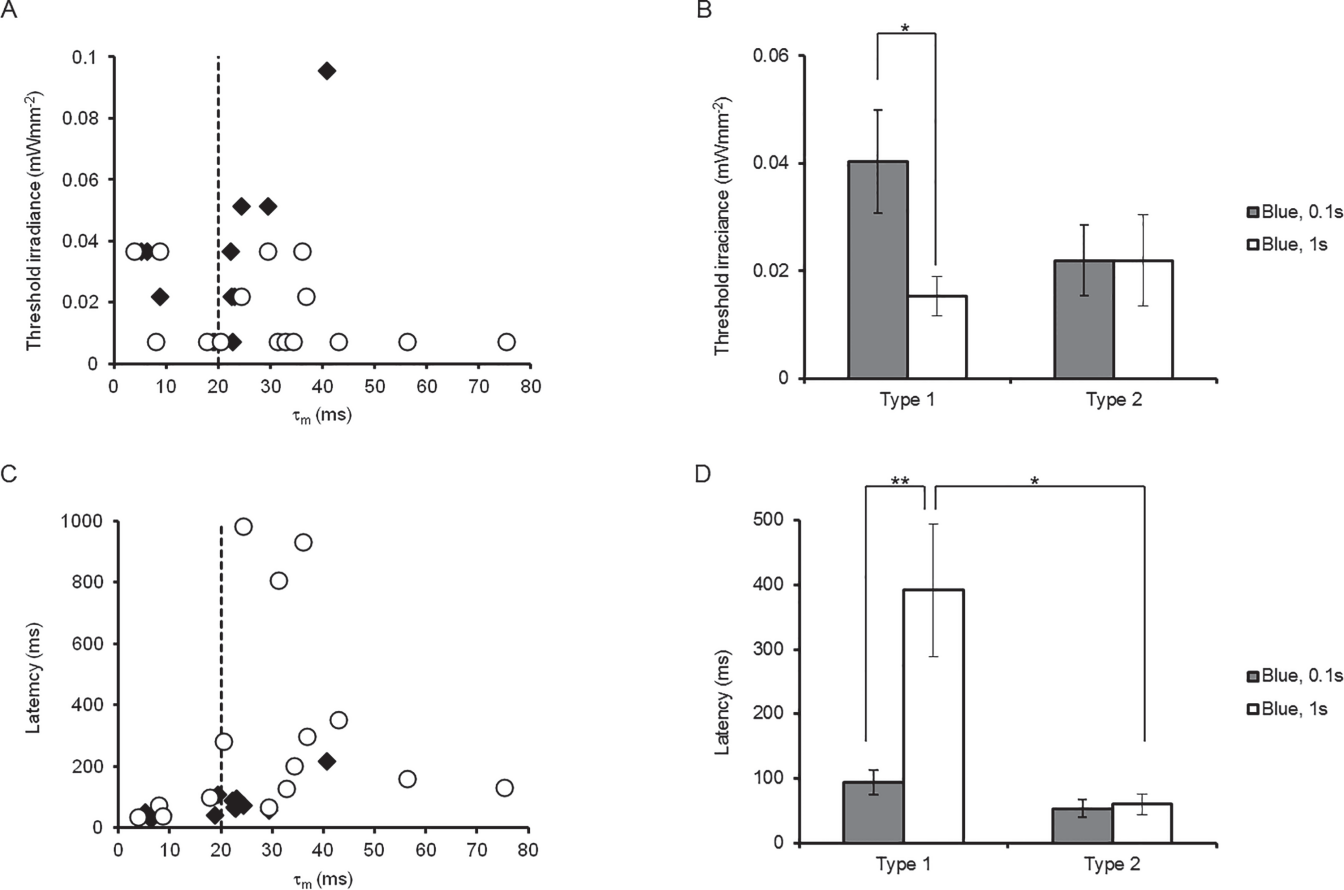
Differential sensitivity to irradiance and duration among neurons. **A,** The threshold irradiance to evoke an action potential was related to the membrane time constant (τ_m_) for the blue LED pulse of either 0.1 s (closed diamonds) or 1 s (open circles). **B,** Reduction of threshold irradiance of the type-1 neurons with prolongation of the pulse duration. **C,** The latency to evoke an action potential was related to τ_m_. **D,** The delayed firing of type-1 neurons with prolongation of the pulse duration. Statistical significance was evaluated with Mann-Whitney *U* test; *, P < 0.05 and **, P < 0.005.

As shown in Fig. 6A, which shows the relationship between the threshold irradiance and τ_m_, the longer irradiation more effectively reduced the threshold irradiance in neurons with larger τ_m,_ but was less effective in neurons with smaller τ_m_. For convenience, the cortical neurons were classified into two groups: type 1 (τ_m_ > 20 ms, 38 ± 4.7 ms, n = 11) and type 2 (τ_m_ < 20 ms, 12 ± 3.1 ms, n = 5) with the significant difference of τ_m_ (P < 0.001). Indeed, the threshold irradiance was reduced by the prolongation of the irradiation time in the case of type-1 neurons, whereas it was less dependent in the case of type-2 neurons (Fig. 6B). However, the reduction of the threshold irradiance was often accompanied by prolongation of the latency to evoke the first action potential in the case of type-1 neurons (Fig. 6C and 6D).

## Discussion

In this study we focused on the amino acid residues C167 and D195 of chimeric ChRs such as ChRWR/C1C2 and ChRFR, respectively counterparts of C128 and D156 of ChR2. Each mutation caused the photocurrent kinetics to become bi-stable with prolongation of the τ_OFF_ by 3–4 orders of magnitude. The conductive state was shut-off by yellow-orange light. Therefore, the conductive state is stabilized by the alanine-replacement of either C167 or D195 of ChRWR or ChRFR. With the difference in the spectrum preference of the basal and conductive states, the photocurrents of these ChR variants were turned on by blue-cyan LED light and shut off by yellow-orange LED light. Although C167 and D195 are respectively provided from TM3 and TM4 of ChR1 in the case of the ChRWR mutants, they were involved in the formation of the DC gate [13], [20]. These results suggest that the arrangements of these residues should be similar between ChR2 and ChRWR, for the latter of which the crystallographic structure was confirmed [24]. Probably, the interaction between C128/167 and D156/195 would be indirect via a water molecule [31]. Alternatively, their arrangements may be changed in the conductive state. This could be solved by future structural studies of the conductive conformation.

Under close inspection the τ_OFF_ of ChRWR-C167A was between those of ChR2-C128A and ChRFR-C167A. The τ_OFF_ of ChRWR-D195A was also between those of ChR2-D156A and ChRFR-D195A. Therefore, the structural difference between the ChR1(TM3-5) and ChR2(TM3-5) backbones appears to minimally influence the interaction within the DC gate and between the DC gate and other components such as T127/166, E123/162 and the Schiff base [31], [32]. Similar mutations were previously characterized in C1V1 chimeric ChR, which consists of TM1-2 of ChR1 and TM3-7 of VChR1 [33]. In this case, the alanine replacement of D195 did not prolong τ_OFF_ and the serine replacement of C167 did so only by 40-fold. The relatively small effect of the DC-gate mutation could be attributed to the structural difference between the ChR2(TM3-7) and VChR1(TM3-7) backbones.

In the present study, it was experimentally demonstrated that the turning-on rate (τ_ON_^−1^) of the photocurrent and the shutting-off rate (τ_OFF_^−1^) of the ion-conducting state is linearly related to the relatively wide range of irradiance (*L*). This is consistent with the prediction that both the transition from basal (P480) to conductive states (P520) and the transition from P520 to P480 is approximated by a single-photon reaction. That is,

$$\tau_{ON}^{\;-1}=\varepsilon_{b}\varphi_{b}L,$$ (1)

and

$$\tau_{OFF}^{\;-1}=\varepsilon_{c}\varphi_{c}L,$$ (2)

where the constants ε_b_ and ε_c_ are respectively molar absorption coefficient equivalents of P480 and P520, and are determinants of the spectral sensitivity of each state. The constants φ_b_ and φ_c_ are respectively quantum yield equivalents of P480 and P520. The φ_b_ is proportional to the probability of a molecule to change its conformation from P480 to P520, whereas the φ_c_ is proportional to the probability of changing from P520 to P480. Each steepness, ε_b_φ_b_ and ε_c_φ_c_, which was experimentally quantified, would give us some insight into differences in the molecular dynamics among ChRs. These values would also be key parameters for predicting the photocurrent kinetics of each ChR as a function of irradiance (*L*) and time.

The steepness (ε_b_φ_b_) of the turning-on rate to irradiance was in the order of ChR2-C128A (640) > ChRWR-C167A (530) > ChRFR-C167A (480) for blue light and ChRWR-C167A (390) > ChRFR-C167A (240) > ChR2-C128A (190) for cyan light. The conserved order of ChRWR-C167A > ChRFR-C167A may be dependent on the difference of φ_b,_ whereas the non-conserved order should be attributed to the difference in ε_b_. Indeed, the C/B ratio of the steepness ([ε_b_ at cyan light]/[ε_b_ at blue light]) suggested that the action spectrum of the basal state was more red-shifted in the order of ChRWR-C167A (0.73) > ChRFR-C167A (0.49) > ChR2-C128A (0.30), as has been observed among non-mutant chimeric ChRs [22]. As C167 has been suggested to interact with retinal with the π-electron system [24], its influence on the spectral shift would be similar among these chimeric ChRs.

In the case of ChR2-C128A, the τ_OFF_^−1^ is experimentally sensitive to the wavelength of light [16], [17]. As φ_c_ is expected to be independent on the wavelength of light, the spectral sensitivity of the ion-conducting state should be investigated conveniently using the O/Y ratio of steepness (ε_c_φ_c_), which is equivalent to [ε_c_ at orange light]/[ε_c_ at red light]. Although the difference in the O/Y ratio was small, it was revealed for the first time that the spectral preference of the conductive state was red-shifted in the order of ChR2-C128A (0.28) > ChRFR-C167A (0.23) > ChRWR-C167A (0.19). Therefore, the order of red shift was different between the ON and OFF responses. This could be attributed to the fact that the influence of apoprotein on the retinal is different between all-*trans* and 13-*cis* configurations, although the precise mechanisms are unknown. It seems to be difficult to investigate the spectral sensitivity of either the basal or conductive state for a broad range of wavelengths because of the overlap among states, P480, P520 and P390 (transient intermediate between P480 and P520) [16].

The steepness (ε_c_φ_c_) of τ_OFF_^−1^ was in the order of ChRWR-C167A (47) > ChRFR-C167A (25) > ChR2-C128A (16) for yellow light and ChRWR-C167A (9.0) > ChRFR-C167A (5.8) > ChR2-C128A (4.4) for orange light. The order of ChRWR-C167A > ChRFR-C167A > ChR2-C128A was thus conserved with different spectra of light, probably because the order would be more dependent on φ_c_ than on ε_c_. This might be attributable to the thermal instability of the ion-conducting state as the magnitude of τ_OFF_^−1^ was in the same order even in darkness (Fig. 2A).

Based on the relationship between τ_OFF_^−1^ and the irradiance (Fig. 3G-3I), the irradiation time necessary to reach full deactivation is predicted to be dependent on the irradiance (S1 Fig.). For example, in the case of ChRFR-C167A, it would be necessary to irradiate over 10 s with the yellow light of 0.01 mWmm^−2^ but over 30 s with orange light of the same strength. The OFF response was less efficient for the irradiance than the ON response, due in part to the fact that the color of light (yellow-orange) was not optimal for absorption by 13-*cis* retinal of the conductive state, as has been suggested previously [16].

Although the amplitude of the photocurrent has been rather simply described as a function of power [16], [17], [18], it actually should be a function of both irradiance and time. As the τ_OFF_^−1^ of each alanine-replaced mutant was negligible during blue/cyan irradiation, the magnitude of the photocurrent can be predicted from the steepness of τ_ON_^−1^ to the irradiance, as shown in Fig. 4G-4I (S2 Fig.). For example, the amplitude of the ChR2-C128A photocurrent becomes almost maximal in 0.1 s with a blue LED light at 0.1 mWmm^−2^. However, irradiation over 1 s is necessary to reach the same amplitude with a blue LED light at 0.01 mWmm^−2^. The ChR2-C128A photocurrent was less sensitive to the cyan LED light and 3.3-fold irradiance was necessary to accomplish the same effect as the blue LED light, as predicted from the slopes of the lines in Fig. 4G. On the other hand the sensitivity difference between the cyan and blue LED light was small in the case of ChRWR-C167A and ChRFR-C167A.

The above consideration allowed us to presume that the threshold irradiance to evoke action potentials would be reduced with prolongation of the irradiation time. When a neuron that expresses ChRFR-C167A has a relatively large τ_m_ (type-1 neuron), its membrane potential is depolarized slowly with an increase of the photocurrent during irradiation. As a result, it evokes an action potential with a relatively small magnitude of irradiation that is ineffective with a short irradiation time. However, in the case of type-2 neurons, which have a smaller τ_m_, the membrane potential was rapidly depolarized to reach the threshold for the action potential. Therefore, prolongation of the irradiation time was less effective to reduce the threshold irradiance. The ChRFR-C167A-expressing neurons were all type 1 (7/7) when the gene was driven by the CaMKIIα promoter. On the other hand, they were either type 1 (4/9) or type 2 (5/9) when the gene was driven by the CAG promoter. Therefore, the type-1 neurons included the excitatory pyramidal neurons, whereas type-2 neurons included the GABAergic interneurons. However, it should be kept in mind that the neuronal responses are also dependent on the metabolic states such as those regulated by neuromodulators because τ_m_ is a function of the membrane resistance (R_m_) and the capacitance (C_m_) (τ_m_ = R_m_ C_m_ in an ideal cell).

These results indicate that the bi-stable variants of ChR were effective in reducing the threshold irradiance, as suggested previously [16], [17], [18], but only with relatively long depolarization. Moreover, the effectiveness was dependent on the neuronal type; more effective for the excitatory pyramidal neurons but less for the smaller interneurons. Even in the absence of any synaptic inputs, the latency to evoke action potentials was variable and rather prolonged. The neuronal membrane potential usually fluctuates *in vivo* with many synaptic inputs. The small and slow photocurrent of bi-stable ChRs generated by the relatively small magnitude of irradiance would thus facilitate the generation of action potentials at each maximal peak of membrane fluctuation. Those bi-stable ChRs such as ChR2-C128A, ChRFR-C167A and ChRWR-C167A would be most suitable for enhancing the intrinsic activity of excitatory pyramidal neurons with irradiation of minimal magnitude. Practically, it would be ideal for neuroscience to photo-activate the neuron of interest with minimal irradiance and maximal temporal precision. The above consideration suggests that the ChRs with larger ε_b_φ_b_, the intrinsic light sensitivity, could effectively depolarize the membrane potential in a relatively short time even with small irradiance. At present, ChRs with various kinetic properties have been obtained by genome mining as well as by molecular modifications [7], [34]. However, the significance of ε_b_φ_b_ has been discussed in only a limited number of papers [35]. It will be necessary to reevaluate the kinetics in the future to optimize ChRs for neuroscience.

## Supporting Information

S1 Fig Predicted OFF-current kinetics with given irradiance according to the relationship shown in Fig. 3. **A–C,** Shut-off by yellow LED light. **A,** The shutting-off rate constant (τ_OFF_^−1^) of ChR2-C128A with the relationship, *y* = 16*x*+0.073. **B,** ChRWR-C167A with the relationship, *y* = 47*x*+0.23. **C,** ChRFR-C167A with the relationship, *y* = 25*x*+0.08. **D-F,** Shut-off by orange LED light. **D,** ChR2-C128A with the relationship, *y* = 4.4*x*+0.058. **E,** ChRWR-C167A with the relationship, *y* = 9.0*x*+0.12. **F,** ChRFR-C167A with the relationship, *y* = 5.8*x*+0.070.
(TIF)

S2 Fig Predicted ON-current kinetics with given irradiance according to the relationship shown in Fig. 4. **A–C,** Activation by blue LED light. **A,** The turning-on rate constant (τ_ON_^−1^) of ChR2-C128A with the relationship, *y* = 640*x*+2.0. **B,** ChRWR-C167A with the relationship, *y* = 530*x*+1.1. **C,** ChRFR-C167A with the relationship, *y* = 480*x*+0.35. **D-F,** Activation by cyan LED light. **D,** ChR2-C128A with the relationship, *y* = 190*x*+0.76. **E,** ChRWR-C167A with the relationship, *y* = 390*x*+1.9. **F,** ChRFR-C167A with the relationship, *y* = 240*x*+1.1.
(TIF)
