# Supplementary figures and images for: Kinetic Evaluation of Photosensitivity in Bi-Stable Variants of Chimeric Channelrhodopsins
Source: PLoS One. 2015 Mar 19;10(3):e0119558. doi: 10.1371/journal.pone.0119558 (PMC4366085; doi:10.1371/journal.pone.0119558)

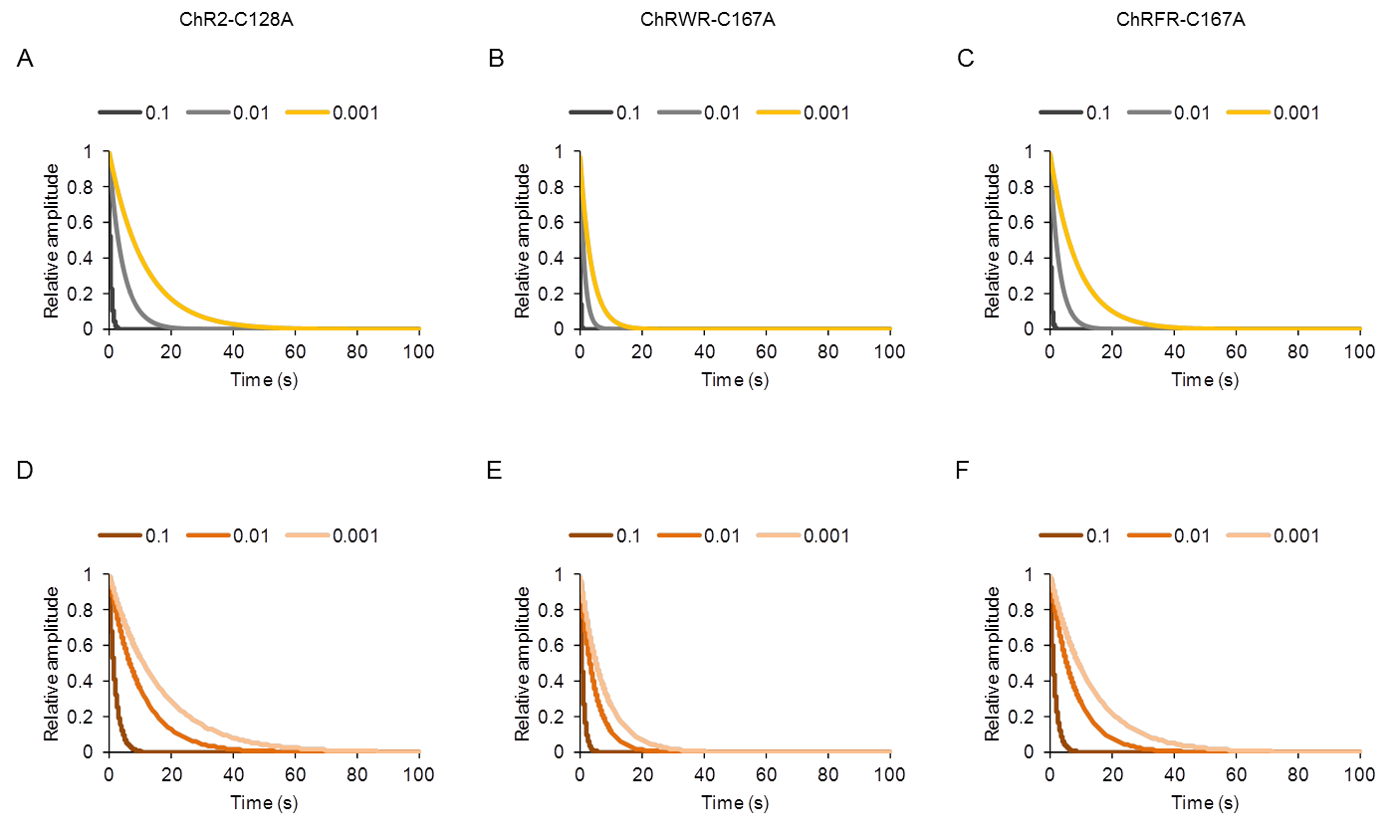

Supplement: S1 Fig — A–C, Shut-off by yellow LED light. A, The shutting-off rate constant (τOFF −1) of ChR2-C128A with the relationship, y = 16x+0.073. B, ChRWR-C167A with the relationship, y = 47x+0.23. C, ChRFR-C167A with the relationship, y = 25x+0.08. D-F, Shut-off by orange LED light. D, ChR2-C128A with the relationship, y = 4.4x+0.058. E, ChRWR-C167A with the relationship, y = 9.0x+0.12. F, ChRFR-C167A with the relationship, y = 5.8x+0.070. (TIF) [file pone.0119558.s001.tif]

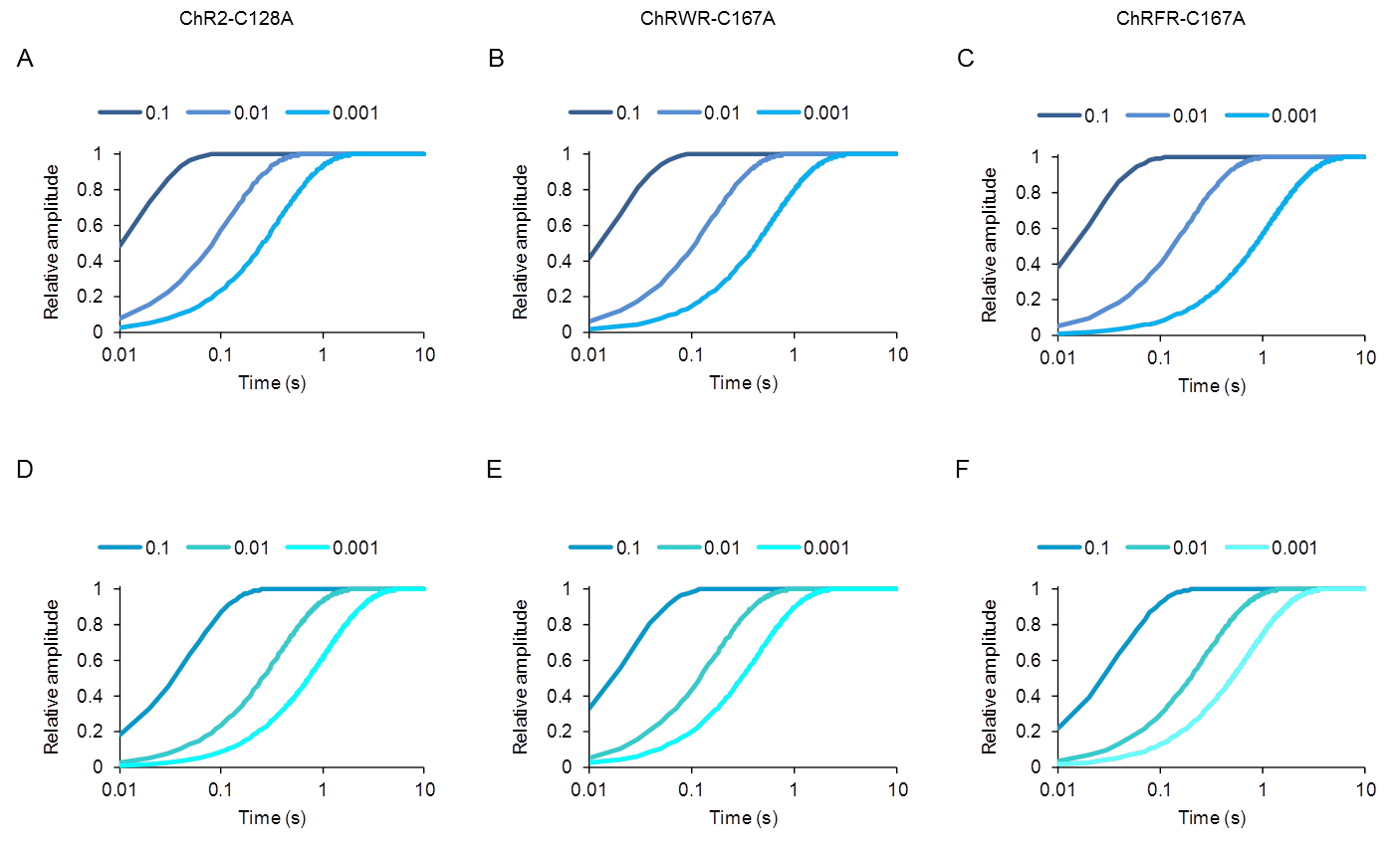

Supplement: S2 Fig — A–C, Activation by blue LED light. A, The turning-on rate constant (τON −1) of ChR2-C128A with the relationship, y = 640x+2.0. B, ChRWR-C167A with the relationship, y = 530x+1.1. C, ChRFR-C167A with the relationship, y = 480x+0.35. D-F, Activation by cyan LED light. D, ChR2-C128A with the relationship, y = 190x+0.76. E, ChRWR-C167A with the relationship, y = 390x+1.9. F, ChRFR-C167A with the relationship, y = 240x+1.1. (TIF) [file pone.0119558.s002.tif]
